# Psychotherapy training in Nepal: views of early career psychiatrists

**DOI:** 10.1192/bji.2020.50

**Published:** 2021-05

**Authors:** Yugesh Rai, Utkarsh Karki, Mariana Pinto da Costa

**Affiliations:** 1 MD, Psychiatry Trainee, Essex Partnership University NHS Trust, UK. Email: raiyogesh39@gmail.com; 2 MD, DM, Consultant Child and Adolescent Psychiatrist, Child and Adolescent Psychiatry Unit, Kanti Children's Hospital, Kathmandu, Nepal; 3 MD, Consultant Psychiatrist, Unit for Social and Community Psychiatry (WHO Collaborating Centre for Mental Health Services Development), Queen Mary University of London, East London NHS Foundation Trust, UK

**Keywords:** Education and training, psychotherapy training, early career psychiatrists, Nepal, low- and middle-income countries

## Abstract

Although training in psychotherapy is an integral part of psychiatry training, standards in psychotherapy training have wide variation across and within countries. Post-graduate psychiatry training has been conducted in Nepal for over two decades, but little is known about its psychotherapy training provisions. An online survey was conducted with early career psychiatrists in Nepal. The findings show that the majority recognised psychotherapy training as important and were eager to pursue further training. However, two-thirds had no access to psychotherapy training opportunities. These results highlight the need to improve access to training in different psychotherapy modalities in Nepal.

Nepal is a low- and middle-income country (LMIC) in South Asia. Postgraduate psychiatry training in Nepal has been in existence for over two decades. Currently, 16 out of 23 training institutes provide postgraduate training in psychiatry. There are five different post-graduate psychiatry training programmes and there is no uniform curriculum or process of evaluation.

Psychotherapy understanding and skills are one of the core elements of psychiatry training and clinical practice. It is considered as an ‘identity’ and a distinction from other medical colleagues. In Europe, a psychotherapy survey conducted among psychiatric trainees in 23 countries showed that 92% considered psychotherapy as key to their professional identity.^1^ Interest in psychotherapy has also been described as a major reason to choose psychiatry as a career for young doctors,^2^ who are eager to learn more about the mind and how they can use themselves more effectively to help patients. There is also a growing body of research into the effectiveness of psychotherapies.^3,4^ International bodies have increasingly called for the inclusion of psychotherapies in the clinical treatment guidelines for different psychiatric disorders and for the incorporation of psychotherapy training in psychiatry training. Therefore, it has become more important than ever for psychiatric trainees to acquire at least basic psychotherapeutic skills to ensure a holistic approach and provide better mental healthcare in the future.

In many Asian countries, psychiatric trainees are strongly encouraged to receive supervision in psychotherapy training. However, this is often not implemented in practice, and there is concern that trainees do not achieve the required competencies during their training. In fact, little is known about the status and experiences of early career psychiatrists (ECPs) in Nepal regarding psychotherapy training.

## Aims

This article aims to: (a) describe the current psychotherapy training status in Nepal; (b) describe ECPs’ experiences of psychotherapy in Nepal; (c) examine how psychotherapy is included in psychiatry training in Nepal; and (d) identify access to psychotherapy training in Nepal.

## Method

An online survey about psychotherapy training was developed by the Section of Early Career Psychiatrists of the World Psychiatric Association (WPA). Participants were early career psychiatrists (ECPs), defined as ‘a psychiatry trainee or a psychiatrist within 7 years after specialising in psychiatry’,^5^ and in this article we report the responses from Nepal.

The questionnaire explored participants’: (a) demographics; (b) opinions about psychotherapy training; and (c) experiences of psychotherapy. The questionnaire was disseminated using Google Forms and emailed to all ECPs in Nepal in November 2018 with one reminder sent. Potential participants approached were all registered medical doctors in Nepal, capable of providing informed consent by responding to the questionnaire, if they decided to take part in the study. This study did not require ethical approval. The questionnaire was made anonymous, and all the responses were unidentifiable.

## Results

A total of 51 ECPs completed the survey (a response rate of 53.7%). The results are summarised in Tables 1–3. The mean age was 31.3 years (± 3.4), and 58.8% were male. More than two-thirds of ECPs reported inclusion of psychotherapy in their psychiatry training. The majority (67.6%) reported that it is mandatory training and four-fifths report that it includes only the theoretical concepts. About one-third had psychotherapy training experience, mostly in cognitive–behavioural therapy (CBT). Experience in interpersonal therapy, family therapy or other therapies was much less common. Among those who had undergone psychotherapy training, only half were satisfied with it. Two-thirds had psychotherapy supervision, but half of them reported that it was optional and that the duration was <50 h. A high proportion of ECPs indicated that psychotherapy training should be mandatory, although only a few of them have undergone personal psychotherapy themselves.

**Table 1:** Demographic details and psychotherapy training status of respondents (*n* = 51)

Demographic details	
Gender	Male: 58.8% (*n* = 30)
Age	Mean 31.3 years (± 3.4)
Job position	Psychiatry trainee: 37.3% (*n* = 19)
	General adult psychiatrist: 58.8% (*n* = 30)
	Child psychiatrist: 3.9% (*n* = 2)
Psychotherapy training in Nepal	
Is psychotherapy included in your psychiatry training?	Yes: 72.5% (*n* = 37)
Mandatory (*v.* Optional)	Mandatory: 67.6% (*n* = 25)
Theoretical (*v.* Practical)	Theoretical: 78.4% (*n* = 29)

**Table 2:** Respondents’ experiences in psychotherapy training (*n* = 51)

Have you done any training in psychotherapy?	
No, have not trained in psychotherapy	64.7% (*n* = 33)
Yes, currently training	29.4% (*n* = 15)
Yes, completed training	5.9% (*n* = 3)
If Yes, which modality? (*n* = 18)	Cognitive–behavioural therapy: 94.4% (*n* = 17)
	Interpersonal therapy: 50% (*n* = 9)
	Family therapy: 38.9% (*n* = 7)
	Psychodynamic therapy: 22.2% (*n* = 4)
	Group therapy: 5.6% (*n* = 1)
	Motivational enhancement therapy: 5.6% (*n* = 1)
Have you undergone personal psychotherapy?	Yes: 3.9% (*n* = 2)
Psychotherapy supervision	
Access to supervision	66.6% (*n* = 12)
Mandatory (*v.* Optional)	Optional: 58.3% (*n* = 7)
Format of supervision (*n* = 12)	Individual: 41.6% (*n* = 5)
	Group: 25% (*n* = 3)
	Mixed: 33.3% (*n* = 4)
Duration of supervision (*n* = 12)	<50 h: 58.3% (*n* = 7)
	50–100 h: 16.7% (*n* = 2)
	>100 h: 25% (*n* = 3)
Satisfaction with psychotherapy training (*n* = 18)	
	Dissatisfied: 22.2% (*n* = 4)
	Satisfied: 50% (*n* = 9)
	Neither satisfied nor dissatisfied: 27.8% (*n* = 5)

**Table 3:** Respondents’ opinions regarding psychotherapy training (*n* = 51)

Should psychotherapy be included in psychiatry training?	Yes: 98% (*n* = 50)
What modality should be included?	Cognitive–behavioural therapy: 96.1% (*n* = 49)
	Family therapy: 82.4% (*n* = 42)
	Interpersonal therapy: 80.4% (*n* = 41)
	Psychodynamic therapy: 56.9% (*n* = 29)
	Psychodrama: 37.3% (*n* = 19)
	Dialectical behaviour therapy: 7.8% (*n* = 4)
Mandatory (*v.* Optional)	Mandatory: 90.2% (*n* = 46)

## Discussion

The main finding of this study was that only one-third of the ECPs in Nepal had received psychotherapy training. This figure might be much lower if we only count colleagues who trained in Nepal, since many respondents trained abroad (particularly in India), with good exposure to psychotherapy training. These shortfalls in psychotherapy training programmes have been highlighted in other countries as well.^1,6^ In the European psychotherapy survey, less than 50% of the trainees had received psychotherapy training.^1^ One possible explanation is that psychotherapy training is affected by the lack of availability of skilled psychotherapists who can train ECPs. Higher training in psychotherapy is not available, and training centres do not have protected time for psychotherapy training.

It is essential to legitimise psychotherapy for successful implementation of formal psychotherapy training programmes in Asian countries as psychotherapy is considered a Western concept.^7^ In Nepal, a major hindrance to psychotherapy practice was patients’ preference for traditional healing, difficulty in accessing therapy, cultural and language barriers, and non-availability of locally adapted tools.^8^ In Thailand, barriers to psychotherapy training identified by ECPs were time constraints, lack of supervisors and absence of incentives for psychotherapy practice.^9^ Providing psychotherapy training in university settings, relating psychoanalysis to Eastern Taoist philosophy (in Singapore) or to Confucian philosophy (in Korea) was particularly helpful for widespread support to implement psychotherapy training programmes.^7^ Therefore, local and cultural adaption of psychotherapies may be required for successful implementation in practice. It is challenging to bridge this learning gap, given the competing demands of mental health services and few formally trained supervisors in psychotherapy. It is imperative to establish a close liaison and collaboration with other professionals, such as clinical psychologists, to initiate and develop innovative training opportunities. Emphasis on the training can be ensured by allocating protected time specifically for psychotherapy training and supervision.

In this survey, more than two-thirds of ECPs in Nepal reported that psychotherapy is a part of psychiatry training and is mandatory. However, four-fifths reported that that training focused mainly on the theoretical aspects. This may be one of the reasons for the lack of competencies in psychotherapy. CBT was the predominant therapy ECPs had experience of in Nepal. Similarly, a recent survey in India demonstrated that training in different types of psychotherapy, such as psychodynamic psychotherapies, rational emotive therapy and dialectical behaviour therapy, was inadequate or low.^6^ A possible explanation is that CBT is the most supervised of therapeutic modalities in most Asian countries^10^ and training in other modalities is not as readily available and accessible.

One of the most important aspects of psychotherapy training is the supervision of psychotherapy cases. Our study found that two-thirds of those who had access to psychotherapy training received supervision. Half of them reported that the supervision was optional, and the duration was less than 50 h. The Indian Psychiatric Society (IPS) Task Force Guidelines on postgraduate psychiatry training have mandated that each trainee achieves a minimum of 50 h of supervised psychotherapy and submits one therapy case for evaluation as part of final year assessment.^11^ For those who had undergone psychotherapy training, about 50% reported that they were satisfied with it.

Importantly, most ECPs in Nepal considered that psychotherapy should be included in psychiatry training, and it was encouraging to find that ECPs in Nepal were highly motivated for psychotherapy training. Most ECPs would like to pursue further training in CBT, family therapy and interpersonal therapy. Psychodrama and dialectical behaviour therapy received the lowest interest, possibly a reflection that training in these modalities is difficult to access. These findings suggest that the postgraduate training curriculum might need to be updated and implemented to prioritise structured psychotherapy training with adequate supervision and thus enhance the training experience. Access to online training resources in different modalities of psychotherapy^12^ can be a feasible short-term option in the Nepalese context to address these unmet training needs of ECPs.

It is difficult to establish whether this study is a true representation of all ECPs’ experiences (as the response rate was 53.7%) and the questionnaire did not ask about the place and year of training/work, to ensure that the responses were anonymous. Future research could be complemented by directly contacting the training programmes in each institution. It would be important to further explore the reasons for satisfaction or dissatisfaction, which was beyond the scope of this questionnaire. We also did not explore who does the psychotherapy supervision, or the frequency and quality of supervision, and these factors warrant exploration in further research.

Psychotherapeutic treatment can be made more accessible to patients by providing psychotherapy training to psychiatric trainees. Despite being challenging, training in psychotherapy will benefit upcoming young psychiatrists, improve patient care and enhance the specialty.

## Conclusion

Current psychiatry training may be inadequate to meet substantial psychotherapy training opportunities for a high proportions of ECPs in Nepal. It is highly encouraging that the majority of ECPs were eager to pursue further psychotherapy training and there is a scope to improve on existing psychotherapy training.

## References

[1] Gargot T, Dondé C, Arnaoutoglou NA, Klotins R, Marinova P, Silva R, How is psychotherapy training perceived by psychiatric trainees? A cross-sectional observational study in Europe. Eur Psychiatry 2017; 45: 136–8. 28756112 10.1016/j.eurpsy.2017.05.030

[2] Bateman A, Holmes J. Psychotherapy training for psychiatrists: hope, resistance, and reality. Psychiatr Bull 2001; 25: 124–5.

[3] Carr A. The Effectiveness of Psychotherapy: A Review of Research. Irish Council of Psychotherapy, 2007.

[4] Munder T, Flückiger C, Leichsenring F, Abbass A, Hilsenroth M, Luyten P, Is psychotherapy effective? A re-analysis of treatments for depression. Epidemiol Psychiatr Sci 2018; 28: 268–74. 30058524 10.1017/S2045796018000355 PMC6998909

[5] Pinto da Costa M. Early Career Psychiatrists – history, 2020 and beyond. World Psychiatry 2020; 19: 127–8. 31922680 10.1002/wps.20712 PMC6953585

[6] Grover S, Sahoo S, Srinivas B, **Tripathi A, Avasthi A.** Evaluation of psychiatry training in India: a survey of young psychiatrists under the aegis of research, education, and training foundation of Indian Psychiatric Society. Indian J Psychiatry 2018; 60: 445–60. 30581210 10.4103/psychiatry.IndianJPsychiatry_334_18 PMC6278221

[7] Ang A. Psychotherapy training in Singapore. Psychiatr Bull 2001; 25: 112–3.

[8] Halder S, Mahato AK. CBT: the Nepalese experience. In Cognitive Behaviour Therapy in Non-Western Cultures (eds F Naeem, D Kingdon): 79–92. Nova Science Publishers, 2012.

[9] Charernhoon T, Phanasathit M. Psychotherapy practices and training experiences: a national survey of young Thai psychiatrists. J Med Assoc Thai 2011; 94(suppl 7): S95–101. 22619914

[10] Alfonso CA, Sutanto L, Zakaria H, **Kalayasiri R, Lukman PR, Elvira SD,** Psychodynamic psychotherapy training in South East Asia: a distance learning program. BJPsych Int 2018; 15: 8–11. 29953132 10.1192/bji.2017.5 PMC6020918

[11] Issac M, Murthy P, Sidana A, **Sharma P, Ghosal M, Subodh BN,** Taskforce Guidelines for Postgraduate Psychiatry Training in India. Indian Psychiatric Society, 2013.

[12] EFPT Psychotherapy Guidebook (EPG) Team. EFPT Psychotherapy Guidebook. PubPub, 2020 (https://epg.pubpub.org).

